# *Plasmodium vivax*: Induction of CD4^+^CD25^+^FoxP3^+^ Regulatory T Cells during Infection Are Directly Associated with Level of Circulating Parasites

**DOI:** 10.1371/journal.pone.0009623

**Published:** 2010-03-10

**Authors:** Lilian Lacerda Bueno, Cristiane Guimarães Morais, Fernanda Fortes Araújo, Juliana Assis Silva Gomes, Rodrigo Corrêa-Oliveira, Irene Silva Soares, Marcus Vinícius Lacerda, Ricardo Toshio Fujiwara, Érika Martins Braga

**Affiliations:** 1 Departamento de Parasitologia, Instituto de Ciências Biológicas, Universidade Federal de Minas Gerais, Belo Horizonte, Brazil; 2 Laboratório de Imunologia Celular e Molecular, Instituto René Rachou, FIOCRUZ, Belo Horizonte, Brazil; 3 Departamento de Morfologia, Instituto de Ciências Biológicas, Universidade Federal de Minas Gerais, Belo Horizonte, Brazil; 4 Departamento de Análises Clínicas e Toxicológicas, Universidade de São Paulo, São Paulo, Brazil; 5 Fundação de Medicina Tropical do Amazonas, Manaus, Brazil; New York University, United States of America

## Abstract

Circulation CD4^+^CD25^+^FoxP3^+^ regulatory T cells (Tregs) have been associated with the delicate balancing between control of overwhelming acute malaria infection and prevention of immune pathology due to disproportionate inflammatory responses to erythrocytic stage of the parasite. While the role of Tregs has been well-documented in murine models and *P. falciparum* infection, the phenotype and function of Tregs in *P. vivax* infection is still poorly characterized. In the current study, we demonstrated that patients with acute *P. vivax* infection presented a significant augmentation of circulating Tregs producing anti-inflammatory (IL-10 and TGF-β) as well as pro-inflammatory (IFN-γ, IL-17) cytokines, which was further positively correlated with parasite burden. Surface expression of GITR molecule and intracellular expression of CTLA-4 were significantly upregulated in Tregs from infected donors, presenting also a positive association between either absolute numbers of CD4^+^CD25^+^FoxP3^+^GITR^+^ or CD4^+^CD25^+^FoxP3^+^CTLA-4^+^ and parasite load. Finally, we demonstrate a suppressive effect of Treg cells in specific T cell proliferative responses of *P. vivax* infected subjects after antigen stimulation with Pv-AMA-1. Our findings indicate that malaria vivax infection lead to an increased number of activated Treg cells that are highly associated with parasite load, which probably exert an important contribution to the modulation of immune responses during *P. vivax* infection.

## Introduction

Malaria is a major worldwide scourge, infecting and killing several millions of individuals each year [1]. Of the species that infect humans, *Plasmodium vivax* and *Plasmodium falciparum* are the two most important human malaria parasites. While deaths by *P. vivax* are rare compared to the *P. falciparum*, there is an increasing number of publications reporting severe disease, including respiratory distress and coma as a result of *P. vivax* infection [2], [3]. Although the worldwide burden of *P. vivax* malaria has not been reliably estimated, the annual infections may range from 132 million to 391 million people [4] and 2.6 billion people living in areas of risk [5]. This disease affects poor people living in least developed and developing countries. Infection by this parasite may result in life-long learning impairment, incapacitating adults for work, with major direct economic consequences due to loss of productivity and depletion of the already meager financial resources [6]. Despite the importance of this disease, representing the most prevalent recurrent malaria [7], the immunological mechanisms associated to the control of parasite levels and disease severity are not fully understood.

Protective cellular immune responses against malaria can be initiated by antigen-presenting cells (e.g. dendritic cells) that ultimately activate specific CD4^+^ and CD8^+^ T cells. The resulting protective Th1-dependent immune responses to blood-stage malaria infection [8] is largely mediated by IFN-γ and TNF-α [9]. These cytokines act synergistically to optimize nitric oxide production [10], which have been associated with parasite killing [11]. Paradoxically, the morbidity of acute malaria is associated with severe immune-mediated pathology due to disproportionate inflammatory responses to the erythrocytic stage of the parasite [12]. The delicate balancing between control of infection and prevention of immunopathology [13] is attributed to CD4^+^CD25^+^FoxP3^+^ regulatory T cells (Tregs), which play an important role in maintaining immune homeostasis and controlling excessive immune responses [14]. These cells have been shown to suppress cellular immune responses through direct contact with immune effector cells and by the production of regulatory cytokines, including TGF-β and IL-10 [15].

Evidences of the role of Treg cells as suppressors of T cell responses in malaria were initially demonstrated in murine models, where these cells have been associated with increased [16], [17] or delayed [18], [19] parasite growth. Higher Treg cell numbers are associated with increased parasite load [20]–[22] and development of human infection caused by *P. falciparum* [23]. A functional deficit of Treg cells, characterized by reduced expression of CTLA-4 (cytotoxic T lymphocyte antigen 4) and FoxP3 (forkhead box P3 transcription factor), was observed in studies involving the Fulani ethnic group that present low susceptibility to clinical malaria by *P. falciparum* [24].

While the role of Tregs in malaria infection has been well-documented in murine models and *P. falciparum* infection, the association of Treg cells and *P. vivax* infection is still poorly understood. A recent study by Jangpatarapongsa and colleagues [25] demonstrated an increase on the number of IL-10-producing Treg cells in *P. vivax*-infected individuals. However, further phenotypic and functional characterization of Tregs cells in vivax malaria is still needed.

In the current study, we describe the augmentation of circulating Treg cells in peripheral blood of *P. vivax*-infected individuals and its possible association with parasite burden. We also show the expression of molecules (CTLA-4 and glucocorticoid-induced tumor necrosis factor receptor - GITR) as well as pro- and anti-inflammatory cytokines associated with suppression by Tregs. Furthermore, the association between Treg subpopulations and parasitemia was evaluated. We also demonstrated these cells have a suppressive effect on *in vitro* T cell proliferative responses of individuals infected with *P. vivax*. Our results point to an increased numbers of activated Treg cells that are significantly associated with parasite load and may exert their function by modulating the immune responses during *P. vivax* infection.

## Materials and Methods

### Study Population and Blood Samples

Samples from 30 patients older than 18 years old with non-complicated *P. vivax* malaria were used in the study. All patients were resident in Manaus, the capital of the Amazonas State (Western Brazilian Amazon). The patients were unrelated outpatients being diagnosed at the Fundação de Medicina Tropical do Amazonas. Fifteen healthy adult blood donors were recruited for the study over the course of several months from Belo Horizonte, Minas Gerais State, Brazil, a non-endemic area for malaria. The study was approved by the Ethics Committee on Research with Humans of Universidade Federal de Minas Gerais (Protocol# ETIC 060/07). Blood was obtained after receiving the signed inform consent.

Venous blood was collected immediately before the beginning of the antimalarial treatment in EDTA and heparin-containing tubes (4 and 32 mL, respectively) and was used to prepare thick smears for microscopy, to extract parasite DNA and for PBMC isolation. Parasitological evaluation was performed by examination of 200 fields at l.000× magnification under oil-immersion. All slides were examined by at least two well-trained microscopists from the Brazilian Ministry of Health. The *P. vivax* mono-infection was confirmed by PCR as previously described [26]. Hemoglobin, hematocrit (HCT) and platelet levels were measured using an automated blood cell counter (ABX Pentra 90; Horiba Diagnostics, Kyoto, Japan) (Table 1). Correlation between platelet counts and the level of parasitemia and hemoglobin level and parasitemia was determined for both infected and control donors (Figure S1A and S1B, respectively).

**Table 1:** Description of the study population by age and hematological parameters (Mean ± SD).

	Individuals	
Characteristics	*Malaria*-infected+ (n = 30)	*Malaria*-naïve (n = 15)
Age mean, years	37.6±12.63	36.43±11.96
Hemoglobin (g/dL)	12.8±1.6*	16.1±1.1
Platelets (cells/mm^3^)	96,600±45,300*	184,400±20,300
Parasitemia (parasites/µL)	4920±3474	0

+ *P. vivax* infection detected by parasitological smears and PCR.

*Statistically different from control group (P<0.0001).

### Isolation of Peripheral Blood Mononuclear Cells

Peripheral blood mononuclear cells (PBMCs) were obtained as previously described [27]. Briefly, cells were isolated from heparinized blood on a density gradient centrifugation (Histopaque®, Sigma Aldrich Co., USA) and were resuspended at a final concentration of 1×10^7 ^cell/mL in RPMI 1640 medium (Invitrogen Co., USA) supplemented with 2 mM of L-glutamine (Sigma), 5% heat-inactivated human AB serum (Sigma) and 6% Antibiotic-Antimycotic solution (Invitrogen).

### Cell Phenotyping by Flow Cytometry and Intracellular Staining

PBMCs were stained using monoclonal antibodies to determine the expression of T regulatory cell markers (CD4, CD25 and FoxP3), and co-expression of GITR (glucocorticoid-induced tumor necrosis factor receptor), CD152/CTLA-4 (Cytotoxic T-Lymphocyte Antigen 4), IFN-γ, IL-17, IL-10 and TGF-β.

Cells were incubated with 2 µL of undiluted monoclonal antibodies (all from BD Pharmingen, USA) conjugated either with fluorescein isothiocyanate (FITC), phycoerythrin (PE) or allophycocyanin (APC) in the dark for 30 min at room temperature. Intracellular staining for FoxP3, CTLA-4 and cytokines was performed using the eBioscience fixation/permeabilization buffer kit following manufacturer's instructions. After incubation, PBMCs were washed twice with 2 mL of phosphate-buffered saline containing 0.01% sodium azide followed by fixation in 200 µL of fixative solution (10 g/L paraformaldehyde, 1% cacodylic acid, 6.65 g/L sodium chloride, 0.01% sodium azide). Phenotypic analyses were performed using a Becton Dickinson FACScalibur flow cytometer and the analysis was performed using the CellQuest software (BD Biosciences, USA) (Figure S2).

### Recombinant Pv-AMA-1

The recombinant protein representing amino acids 43 to 487 of Pv-AMA-1 [28], [29] was expressed in *Escherichia coli* BL21(DE3) at 37°C for 3 h by adding 0.1 mM isopropyl-1-thio-β-D-galactopyranoside (IPTG, Invitrogen), as previously described [27]. Pv-AMA-1 antigen was tested to determine the presence of Gram-negative bacterial endotoxin using a chromogenic Limulus Amebocyte Lysate test (QCL-1000, Cambrex, USA) according to the manufacturer's instruction, and was found to be a non significant source of endotoxins (levels lower than detection limit of 5 EU/mL).

### Analysis of the Effect of CD4^+^CD25^+^ T Cells on *In Vitro* Cellular Proliferation to Pv-AMA-1

For the analysis of suppression activity, CFDA-SE labeled PBMCs from *P. vivax* infected individuals that presented positive proliferative responses for *P. vivax* AMA-1 antigen were co-incubated with autologous CD4^+^CD25^+^ isolated cells. CFDA-SE labeling of freshly isolated PBMCs (10^6^ cells/mL in PBS/1% BSA) was performed by incubation of 0.4 µM CFDA-SE (Molecular Probes, USA) for 10 minutes at room temperature. CD4^+^CD25^+^ lymphocytes (T regulatory cells) were purified from PBMCs by magnetic bead separation using a QuadroMACS cell separator (Miltenyi Biotec, USA). CD4^+^ T cells (>97% purity) were purified by using a T cell isolation kit (Miltenyi Biotec, USA), and CD4^+^CD25^+^ T cells were enriched by a single-step positive selection using anti-CD25 microbeads (Miltenyi Biotec). All microbead isolations followed the manufacturer's instructions. The purified CD4^+^CD25^+^ T regulatory cells were incubated at different concentrations (1∶2, 1∶5 and 1∶10 ratios) with autologous CFDA-SE-labeled PBMCs pulsed with *P. vivax* AMA-1 (1 µg/well), for 96 hours at 37^o^C and 5% CO_2_ atmosphere.

The cell proliferative response of PBMCs co-incubated with CD4^+^CD25^+^ T regulatory cells was assessed using a Becton Dickinson FACScan flow cytometer. Data on 5×10^4^ lymphocytes were acquired and the analysis was performed using the CellQuest software (BD Biosciences, USA). The results are expressed as CFDA-SE proliferation ratio, calculated by the proliferative response observed in co-cultures of Pv-AMA-1-stimulated PBMCs with CD4^+^CD25^+^ T cells over Pv-AMA-1-stimulated PBMCs only. Additional controls using antigen only or mitogen (PHA, Sigma, USA) were also included. CFDA-SE labeling of freshly isolated PBMCs (10^6^ cells/mL in PBS/1% BSA) was performed by incubation of 0.4 µM CFDA-SE (Molecular Probes, USA) for 10 minutes at room temperature. Cells were washed and set up as described above.

### Statistical Analysis

The one-sample Kolmogorov-Smirnoff test was used to determine whether variability followed a normal distribution pattern. P values were determined by two-tailed Mann-Whitney U test. Correlation analysis was performed using Spearman rank correlation. A P value<0.05 was considered significant. All statistics were carried out using Prism 5.0 for Windows (GraphPad Software, Inc.) software.

## Results

### Regulatory T Cell Frequency Is Elevated in *P. vivax* Infected Donors

**Figure 1:**
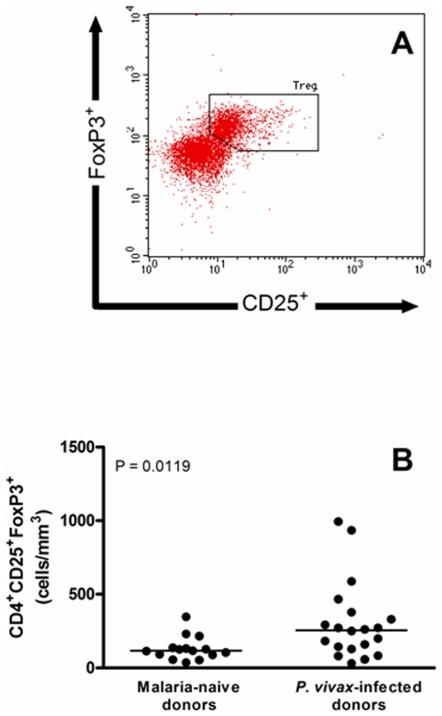
Flow cytometric analysis of regulatory T cells. (A) CD25 and FoxP3 expression in gated CD4^+^CD3^+^ lymphocytes. Dot plot show a representative data of 35 donors examined. (B) Absolute numbers of circulating CD4^+^CD25^+^FoxP3^+^ regulatory T cells in malaria-naïve and *P. vivax*-infected donors (n = 15 and 20, respectively). Absolute numbers (cells/mm^3^) are indicated on Y-axis and lines represent median. Statistical differences were detected using Mann-Whitney U test and are indicated on the graph with significant P values.

Regulatory T cells were identified by flow cytometry as CD3^+^CD4^+^ T cells expressing both CD25 and FoxP3 markers (Figure 1A) and are reported as absolute numbers of cells per mm^3^. Our data clearly show that number of Treg cells was significantly increased in *P. vivax*-infected subjects (median = 256.2 cells/mm^3^) when compared to malaria-naïve donors (median = 116.5 cells/mm^3^) (P = 0.0119, Figure 1B). Further analysis show that frequency of Treg cells (demonstrated by proportion of positive cells) were also elevated in *P. vivax*-infected subjects (P = 0.0009, Figure S3A). Although the difference of both absolute number and proportion of CD4^+^CD25^+^FoxP3^+^ cells between infected and control donors, the level of expression of FoxP3 was similar in both groups (P = 0.0833, data not shown). Since the increased absolute number of CD4^+^CD25^+^FoxP3^+^ cells would reflect a possible augmentation of total CD4^+^ lymphocytes, the number of total CD4^+^ cells was also analyzed. No difference in the absolute number of this lymphocyte population was observed between malaria infected and naïve individuals (data not shown).

### Peripheral Blood CD4^+^CD25^+^FoxP3^+^ Subpopulations Are Also Augmented in *P. vivax* Infected Donors

**Figure 2:**
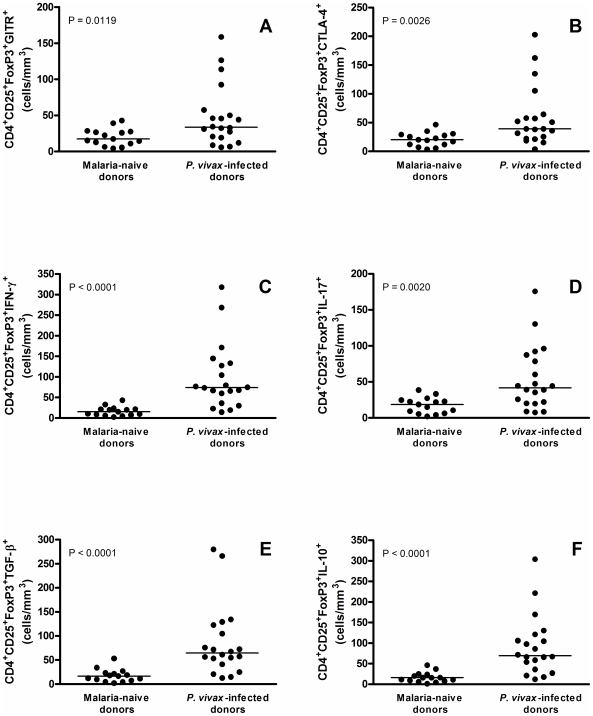
Flow cytometric analysis of surface markers (CTLA-4 and GITR) and cytokines (IFN-γ, IL-17, TGF-β, and IL-10) in CD4^+^CD25^+^FoxP3^+^ regulatory T cells in malaria-naïve and *P. vivax*-infected donors (n = 15 and 20, respectively). Results were expressed as absolute numbers of cells expressing (A) GITR, (B) CTLA-4, (C) IFN-γ, (D) IL-17, (E) TGF-β, and (F) IL-10. Absolute numbers (cells/mm^3^) are indicated on Y-axis and lines represent median. Statistical differences were detected using Mann-Whitney U test and are indicated on the graphs with significant P values.

Once observed the elevated number of Treg cells in the peripheral blood of infected donors, we further characterized this cell population by evaluating the expression of molecules and cytokines associated with cell modulation. Surface expression of the GITR molecule and intracellular expression of CTLA-4 and cytokines were evaluated by flow cytometry. *P. vivax*-infected individuals presented a significant increase of circulating GITR^+^ (P = 0.0119, Figure 2A) and CTLA-4^+^ Treg cells (P = 0.0026, Figure 2B), when compared to malaria-naïve donors. Flow cytometric analysis also showed a significant increase on CD4^+^CD25^+^FoxP3^+^ T cells producing IFN-γ (P<0.0001), IL-17 (P = 0.0020), TGF-β (P<0.0001) and IL-10 (P<0.0001) (Figures 2C to 2F) in malaria-infected individuals. Similar results were observed when proportion of cells expressing CTLA-4^+^, TGF-β, IL-10, IFN-γ, and IL-17 were analyzed (Figures S3C to S3G, P<0.05 for all). No differences in the proportion of GITR^+^ between infected and control individuals was observed (P = 0.3866, Figure S3B).

### Expression of CTLA-4, GITR, TGF-β, IL-10, IFN-γ and IL-17 Is Upregulated in CD4^+^CD25^+^FoxP3^+^ Cells from *P. vivax* Infected Donors

**Figure 3:**
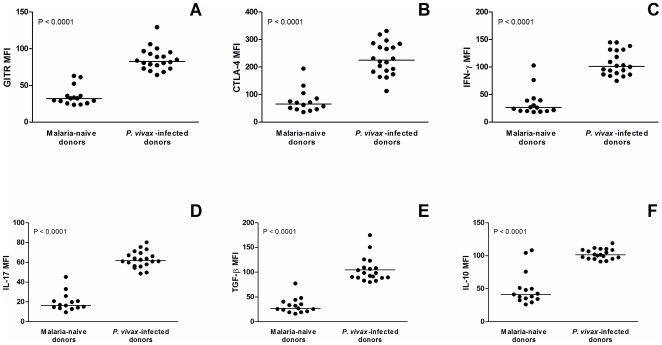
Expression of surface markers (CTLA-4 and GITR) and cytokines (IFN-γ, IL-17, TGF-β, and IL-10) on CD4^+^CD25^+^FoxP3^+^ regulatory T cells in malaria-naïve and *P. vivax*-infected donors (n = 15 and 20, respectively). Results were expressed as median intensity of fluorescence (lines represent median) for (A) GITR, (B) CTLA-4, (C) IFN-γ, (D) IL-17, (E) TGF-β, and (F) IL-10 on CD4^+^CD25^+^FoxP3^+^ cells. Statistical differences were detected using Mann-Whitney U test and are indicated on the graphs with significant P values.

The expression of analyzed surface and intracellular markers was determined by median intensity of fluorescence in order to obtain the absolute expression level per cell basis. Following the increase in the absolute numbers of Treg subpopulations, *P. vivax* infected donors presented a significant higher expression level of all markers tested (CTLA-4, GITR, TGF-β, IL-10, IFN-γ and IL-17) on CD4^+^CD25^+^FoxP3^+^ cells when compared to those observed on malaria-naïve subjects (P<0.0001 for all, Figure 3).

### Absolute Number of Treg Cells Correlates with Parasite Burden in *P. vivax*-Infected Individuals

**Figure 4:**
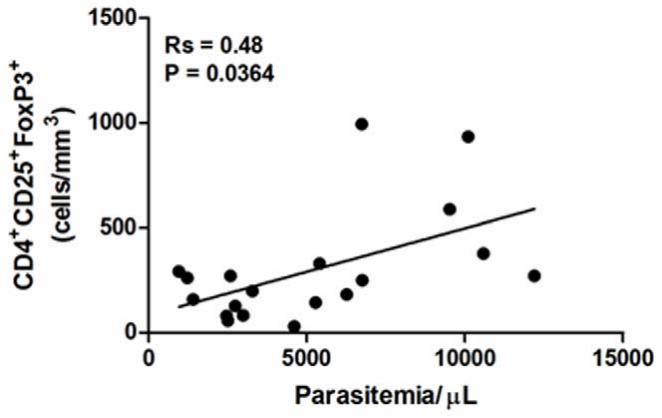
CD4^+^CD25^+^FoxP3^+^ regulatory T cells are directly correlated with parasite burden. The relationship between absolute numbers of CD4^+^CD25^+^FoxP3^+^ regulatory T cells and degree of parasitaemia among 20 patients with *Plasmodium vivax* malaria was examined using Spearman rank correlation.

**Figure 5:**
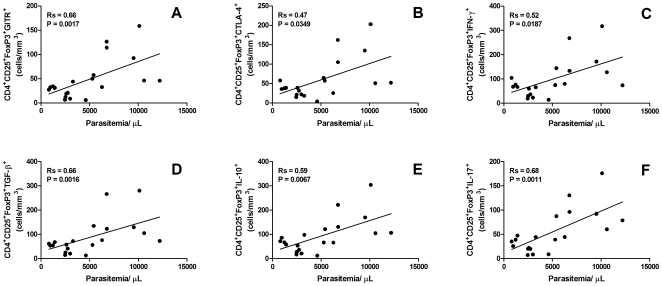
Correlation of CD4^+^CD25^+^FoxP3^+^ regulatory T cell co-expressing (A) GITR, (B) CTLA-4, (C) IFN-γ, (D) TGF-β, (E) IL-10, and (F) IL-17 and degree of parasitaemia among 20 patients with *Plasmodium vivax* malaria. Statistical significance was determined by Spearman rank correlation.

The significantly higher absolute number of circulating CD4^+^CD25^+^FoxP3^+^ cells observed in parasitized donors (Figure 1) suggested that this cell population may have an important role during malaria infection. To further evaluate whether there is a direct correlation between these two variables, the absolute numbers of CD4^+^CD25^+^FoxP3^+^ Treg cells were correlated with the levels of parasitemia (Figure 4). The data clearly shows that the number of peripheral Treg cells increases with the level of parasitemia (Rs = 0.48, P = 0.0364) in *P. vivax* infected individuals. Similar significant correlations were also observed between parasitemia and absolute numbers of Treg expressing GITR (Rs = 0.66, P = 0.0017), CTLA-4 (Rs = 0.47, P = 0.0349), IFN-γ (Rs = 0.52, P = 0.0187), TGF-β (Rs = 0.66, P = 0.0016), IL-10 (Rs = 0.59, P = 0.0067) and IL-17 (Rs = 0.68, P = 0.0011) (Figure 5). These data may imply a direct association between increased parasite number, CTLA-4 and GITR expression and possibly cytokine production.

### CD4^+^CD25^+^ Treg Cells Regulate Antigen-Specific PBMC Proliferation in *P. vivax*-Infected Individuals

**Figure 6:**
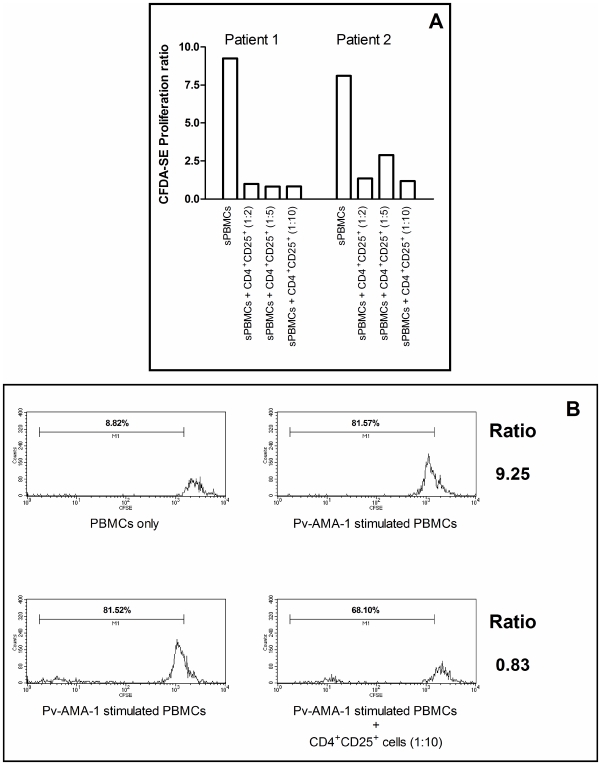
Indirect suppression elicited by CD4^+^CD25^+^ T regulatory cells from *P. vivax*-infected individuals. (A) CFDA-SE Proliferation ratio of Pv-AMA-1- stimulated PBMCs (sPBMCs) and sPBMCs co-cultured with different proportions of autologous CD4^+^CD25^+^ lymphocytes (1∶2, 1∶5 and 1∶10, CD4^+^CD25^+^ cells: sPBMCs). Results are expressed for two malaria-infected donors who presented positive proliferative response after Pv-AMA-1 stimulation. (B) Representative FACS histogram plots for 1 out 2 donors with positive proliferative response after Pv-AMA-1 stimulation showing CFDA-SE staining after antigen stimulation and co-culturing with CD4^+^CD25^+^ T cells. CFDA-SE Proliferation ratio for sPBMCs was calculated by proliferative response observed in Pv-AMA-1-stimulated PBMCs (indicated by positivity for CFDA-SE) divided by basal proliferative response of non-stimulated cells (PBMCs only). CFDA-SE Proliferation ratio for co-cultured cells were calculated by proliferative response observed in Pv-AMA-1-stimulated PBMCs with CD4^+^CD25^+^ T cells divided by proliferative response observed in Pv-AMA-1-stimulated PBMCs only.

In order to determine the possible effect of Treg cells on the immune response during malaria infection, a functional assay was designed to evaluate whether CD4^+^CD25^+^FoxP3^+^ Treg cells can modulate the *in vitro* immune response to parasite antigen. When isolated CD4^+^CD25^+^ T cells were added to the *in vitro* cultures (independent of cell ratio) of Pv-AMA-1-stimulated PBMCs obtained from two individuals (previously selected due to positive *in vitro* proliferative responses after antigen stimulation) a significant reduction on the cell proliferative response elicited by the recombinant Pv-AMA-1 antigen was observed (Figure 6). These results suggest that these cells do have the capacity to modulate the *in vitro* response at least to this *P. vivax*-specific antigenic preparation. No significant differences were observed in cultures stimulated with PHA for all tested individuals (data not shown).

## Discussion

CD4^+^CD25^+^FoxP3^+^ T cells, also known as T regs, play an important role maintaining immune homeostasis and controlling excessive immune responses [14]. These cells suppress cellular immune responses through direct contact with immune effector cells and by production of regulatory cytokines, including TGF-β and IL-10 [15]. Over the past four decades, since its first description in the early 1970's [30], [31], several studies have focused to describe the role of Tregs in infectious diseases (reviewed in [15], [32]), including tuberculosis [33], hepatitis C [34], leishmaniasis [35], [36], helminthiasis [37]–[40] and malaria [16]–[20], [22], [41]–[44]. Although consistent evidence based on experimental models and human *P. falciparum* infection suggest that Tregs may contribute to the onset of infection, the role of these cells during malaria and the possible mechanisms of regulation are not yet fully elucidated. Furthermore, the association of Treg cells and *P. vivax* infection is still poorly understood.

In the current study, we initially showed that *P. vivax*-naturally infected individuals present a significant augmentation of circulating Treg cells in peripheral blood, as previously demonstrated in murine and *P. falciparum* infections [20], [41], [42]. Of note, while absolute number and proportion of CD4^+^CD25^+^FoxP3^+^ cells is higher in malaria infected individuals, no differences were observed for number/proportion of CD4^+^ lymphocytes. The expansion of Tregs in the above settings has been significantly associated with increased or delayed parasite growth [16]–[19] as well as with increased parasite load [20]–[22] and development of clinical malaria [23]. Indeed, herein we also observe a positive correlation between absolute numbers of Treg cells and parasite burden in *P. vivax* malaria. Although the number of peripheral Treg cells increases according to the level of parasitemia, the relationship between number of parasites and host's regulatory T cell activity is still not clear. However, it is possible that increased Treg activity may trigger modulation of host immune response and consequently predisposes to parasite survival and/or failure in the control of parasite multiplication. On the other hand, increased Treg responses might also account for limitation of exacerbated infection-induced pathology, which would be lately beneficial to the host.

A variety of potential mediators of Treg activity that could contribute to the suppression of the host's immune response have been identified, including GITR [45], [46], CTLA-4 [47], FoxP3 [48], [49], and the anti-inflammatory cytokines IL-10 and TGF-β [50]–[52]. Our results show a significant increase of circulating CD4^+^CD25^+^FoxP3^+^GITR^+^ and CD4^+^CD25^+^FoxP3^+^CTLA-4^+^ lymphocytes in *P. vivax-*infected donors, which was further correlated with level of parasitemia observed in the same individuals. Interestingly, a higher expression of these markers on per cell basis was also seen in *P. vivax-*infected donors. Both GITR and CTLA-4 molecules are constitutively expressed on cell surface of natural Tregs [32] and are regulated by FoxP3 expression [53], [54]. Initial studies related to the effects of GITR-signaling on Treg cells indicated that interaction of this receptor with agonist antibody or GITR ligand (GITRL) lead to an apparent abrogation of suppressive activity of Tregs [45], [46], [55]. However, although not essential for the T cell suppressor activity [54], the engagement of GITR promotes proliferation of Tregs [54], [56] and potential enhancement of their suppressive function [55]. Nonetheless, the augmentation of circulating number of CD4^+^CD25^+^FoxP3^+^ co-expressing GITR in *P. vivax* infected subjects might partially reflect the expansion of Tregs observed in those individuals. In this context, we suggest that proliferation of Treg cells in these individuals is elicited by GITR expression, which is possibly upregulated by FoxP3 expression. Although the effect of GITR signaling in Treg cells of *P. vivax* infected donors was not established, evidences from murine malaria suggest that infection alter GITR signaling in Tregs, and this eventually contributes to the escape of parasites from host T cell immunity [57].

The inhibitory receptor CTLA-4 presents partial homology to CD28 molecule and interacts to the same ligands, CD80 and CD86, with a much higher affinity [58]. The suppressive effect of CTLA-4 is associated with the reduced IL-2 production and IL-2 receptor expression, and by arresting T cells at the G1 phase of the cell cycle [59], [60]. Moreover, CTLA-4 expressing Treg cells induce the expression of the enzyme indoleamine 2,3-dioxygenase (IDO) by antigen-presenting cells which degrades tryptophan, and the lack of this essential amino acid inhibits T cell activation and promotes T cell apoptosis [61]. An indirect evidence of the role of this receptor in experimental malaria has been demonstrated by the *in vivo* blockade of CTLA-4 using specific monoclonal antibody, leading to an exacerbation of *P. berghei*-mediated immunopathology [19], [62]. Although the potential functional role of CTLA-4 in regulatory T cell activity remains controversial [63], the increased number of CTLA-4^+^ Treg cells in *P. vivax-*infected donors suggests that regulation may occur through direct contact between antigen-presenting cells and Tregs after engagement of the CTLA-4 pathway. Moreover, since the number of CTLA-4^+^ Tregs is positively associated with parasitemia, it may suggest the involvement of this receptor on the outcome of human malaria. A reduced expression of CTLA-4 was observed in the Fulani ethnic group, contributing to their lower susceptibility to malaria infection and suggesting that alterations in the maturation process of Treg in Fulani individuals may also account for the generation of a lower number of Treg [24].

In this study, patients with acute *P. vivax* infection presented a significant augmentation of CD4^+^CD25^+^FoxP3^+^ cells producing the anti-inflammatory cytokines IL-10 and TGF-β, which also correlated positively with parasite burden. Both cytokines exert a critical role on the regulation of type-1 response in experimental malaria [21] and contribute to Treg cell suppressive activity *in vivo* [64]. This regulatory activity may limit the malaria-induced inflammation, therefore preventing the severity of clinical malaria. However, although IL-10 and TGF-β are naturally produced by Tregs [32] and are required to induce FoxP3 expression [65], it is not clear whether or how parasite replication might influence the production of these cytokines. Conversely, we also observed an increased number of circulating CD4^+^CD25^+^FoxP3^+^ T cells that produce IFN-γ and IL-17 in peripheral blood of *P. vivax-*infected donors, which also correlated with the levels of parasitemia. Recently, Scholzen and colleagues [65] demonstrated the intracellular production of IFN-γ, IL-4 and IL-17, by CD4^+^CD25^+^ Tregs with intermediate (also described as effector T cells) rather than high expression of FoxP3 molecule, suggesting the presence of different subsets of T regulatory cells during malaria. In our study, however, we did not observe any correlation between Foxp3 expression levels and production of all intracellular cytokines assessed (data not shown). Nevertheless, the role of these pro-inflammatory cytokines in malaria-elicited Tregs still remains to be addressed.

Based in our results, we show that malaria-infected individuals present an increased amount of activated Treg cells in the peripheral blood, observed by increased numbers of Treg subpopulations expressing cell surface molecules and mediators associated with suppression of immune responses. Moreover, we show that the same malaria-infected donors had an increased expression of all markers tested. These results might partially explain the reduction of antigen-specific proliferative responses previously demonstrated in individuals infected with malaria [66], and higher plasmatic IL-10 levels in acute *P. vivax* infection [25]. Indeed, evidence from functional assays show that augmentation of CD4^+^CD25^+^ T cells abrogates antigen-specific proliferation of PBMCs from infected individuals, suggesting the role of Tregs during malaria infection. Of note, the ability of Treg cells to abrogate *P. falciparum*-induced cell proliferation in the Fulani population is directly associated to a reduced expression of genes related to suppressive activity. Depletion of Treg cells in Fulani individuals did not significantly increase the proliferation of PBMCs in response to *P. falciparum* antigens, whereas it restored an optimal response to the same antigens in the sympatric highly susceptible Mossi population [24]. Finally, we show that an increased number of circulating Tregs in acute infected individuals is associated with parasite load. In fact, regulatory T cells might limit infection-mediated pathology but also compromise clearance of malaria-infected red blood cells [21]. Whether the function of Tregs is beneficial to the host or to parasite remains to be elucidated. Further studies are still required to support the association of regulatory T cells and immunosupression in a diverse *P. vivax* infection clinical presentation.

## Supporting Information

Figure S1 Correlation between hemoglobin levels (A) and platelet counts (B) and the degree of parasitaemia among patients with *Plasmodium vivax* malaria. Statistical significance was determined by Spearman rank correlation.
(0.10 MB TIF)

Figure S2 FACS analysis. Representative FACS histogram plots for 1 out 35 donors expressing (A) GITR, (B) CTLA-4, (C) IFN-γ, (D) IL-17, (E) TGF-β, and (F) IL-10 in CD4^+^CD25^+^FoxP3^+^ regulatory T cells in malaria-naïve and *P. vivax*-infected donors. CD4^+^CD25^+^FoxP3^+^ cells were initially gated according to Figure 1A. Histogram plots were used to determine the percentage of positive cells and median intensity of fluorescence (MFI) for each stain (M1 indicates positive population).
(0.13 MB TIF)

Figure S3 Flow cytometric analysis of regulatory T cells indicating proportion of total and subpopulations of Treg cells. Results are expressed as percentage of positive cells for (A) CD4^+^CD25^+^FoxP3^+^, (B) CD4^+^CD25^+^FoxP3^+^GITR^+^, (C) CD4^+^CD25^+^FoxP3^+^CTLA-4^+^, (D) CD4^+^CD25^+^FoxP3^+^TGF-β^+^, (E) CD4^+^CD25^+^FoxP3^+^IL-10^+^, (F) CD4^+^CD25^+^FoxP3^+^IFN-γ^+^, and (G) CD4^+^CD25^+^FoxP3^+^IL-17^+^ in malaria-naïve and *P. vivax*-infected donors (n = 15 and 20, respectively). Proportions of positive cells (%) are indicated on Y-axis and lines represent median. Statistical differences were detected using Mann-Whitney U test and are indicated on the graphs with significant P values.
(0.21 MB TIF)
