# Supplementary figures and images for: Plasmodium vivax: Induction of CD4+CD25+FoxP3+ Regulatory T Cells during Infection Are Directly Associated with Level of Circulating Parasites
Source: PLoS One. 2010 Mar 10;5(3):e9623. doi: 10.1371/journal.pone.0009623 (PMC2835751; doi:10.1371/journal.pone.0009623)

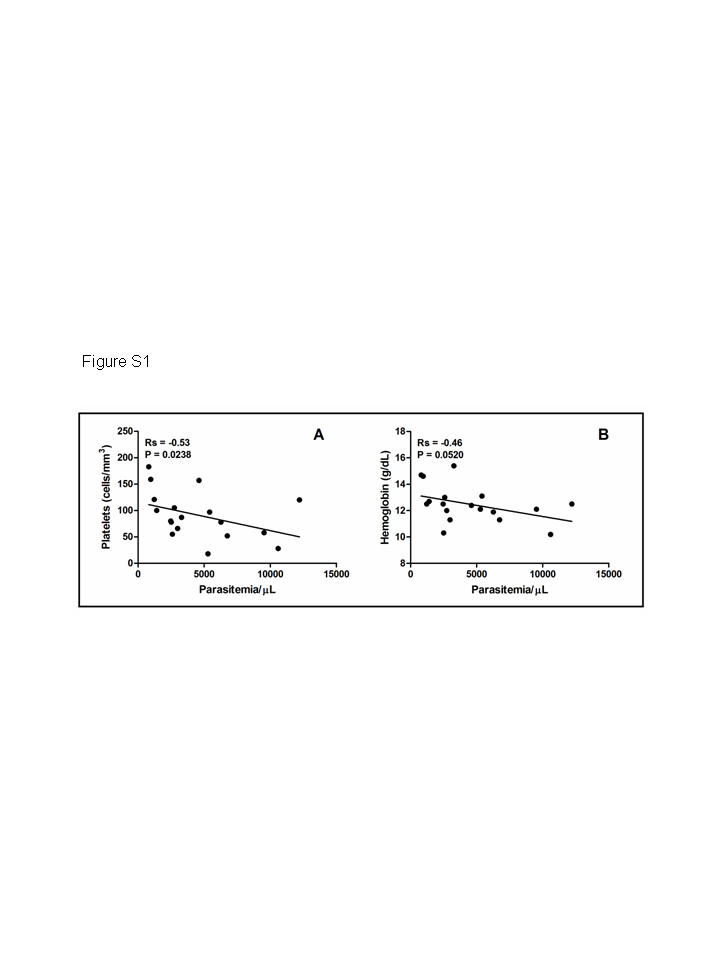

Supplement: Figure S1 — Correlation between hemoglobin levels (A) and platelet counts (B) and the degree of parasitaemia among patients with Plasmodium vivax malaria. Statistical significance was determined by Spearman rank correlation. (0.10 MB TIF) [file pone.0009623.s001.tif]

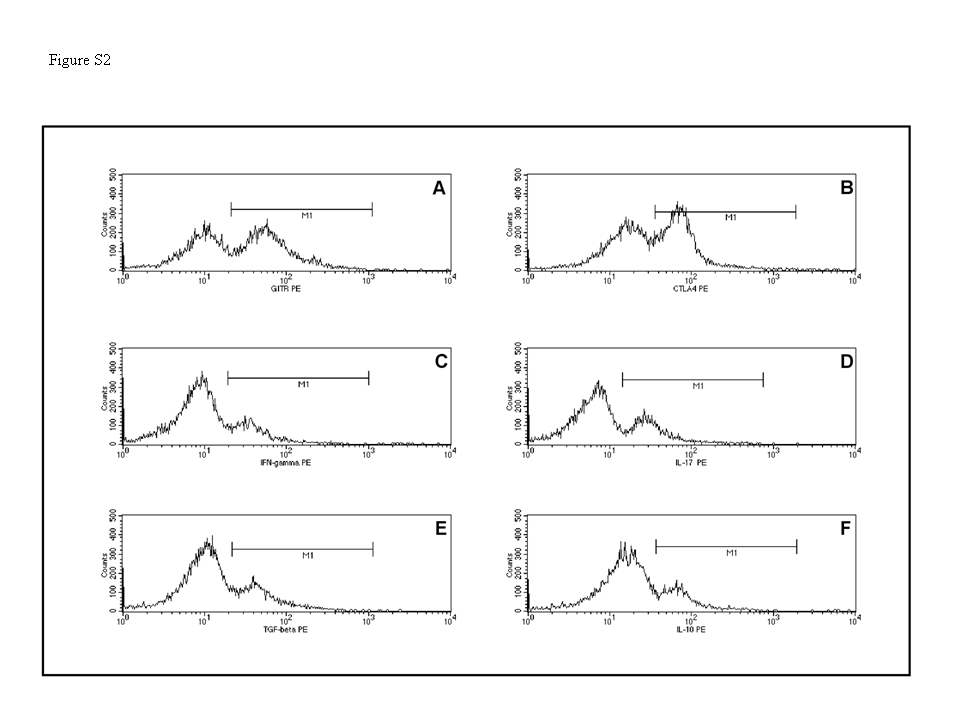

Supplement: Figure S2 — FACS analysis. Representative FACS histogram plots for 1 out 35 donors expressing (A) GITR, (B) CTLA-4, (C) IFN-γ, (D) IL-17, (E) TGF-β, and (F) IL-10 in CD4+CD25+FoxP3+ regulatory T cells in malaria-naïve and P. vivax-infected donors. CD4+CD25+FoxP3+ cells were initially gated according to Figure 1A. Histogram plots were used to determine the percentage of positive cells and median intensity of fluorescence (MFI) for each stain (M1 indicates positive population). (0.13 MB TIF) [file pone.0009623.s002.tif]

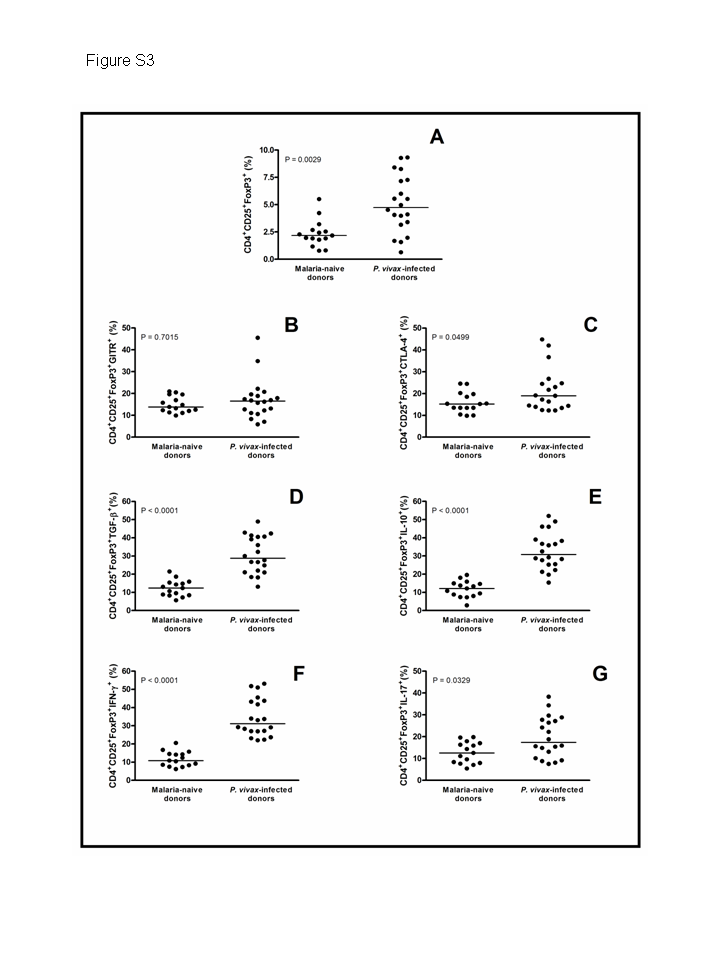

Supplement: Figure S3 — Flow cytometric analysis of regulatory T cells indicating proportion of total and subpopulations of Treg cells. Results are expressed as percentage of positive cells for (A) CD4+CD25+FoxP3+, (B) CD4+CD25+FoxP3+GITR+, (C) CD4+CD25+FoxP3+CTLA-4+, (D) CD4+CD25+FoxP3+TGF-β+, (E) CD4+CD25+FoxP3+IL-10+, (F) CD4+CD25+FoxP3+IFN-γ+, and (G) CD4+CD25+FoxP3+IL-17+ in malaria-naïve and P. vivax-infected donors (n = 15 and 20, respectively). Proportions of positive cells (%) are indicated on Y-axis and lines represent median. Statistical differences were detected using Mann-Whitney U test and are indicated on the graphs with significant P values. (0.21 MB TIF) [file pone.0009623.s003.tif]
